# Prognostic impact of residual lateral lymph node metastasis after neoadjuvant (chemo)radiotherapy in patients with advanced low rectal cancer

**DOI:** 10.1002/bjs5.50194

**Published:** 2019-07-25

**Authors:** T. Akiyoshi, S. Toda, T. Tominaga, K. Oba, K. Tomizawa, Y. Hanaoka, T. Nagasaki, T. Konishi, S. Matoba, Y. Fukunaga, M. Ueno, H. Kuroyanagi

**Affiliations:** ^1^ Gastroenterological Centre, Department of Gastroenterological Surgery Cancer Institute Hospital, Japanese Foundation for Cancer Research Tokyo Japan; ^2^ Department of Gastroenterological Surgery Toranomon Hospital Tokyo Japan; ^3^ Department of Biostatistics, Graduate School of Medicine The University of Tokyo Tokyo Japan

## Abstract

**Background:**

There is a lack of large studies focusing on the prognostic significance of lateral lymph node (LLN) metastasis following LLN dissection (LLND) in rectal cancer. The aim of this study was to evaluate the prognostic impact of LLN metastases on survival of patients with advanced low rectal cancer.

**Methods:**

Consecutive patients with locally advanced, but not metastatic, extraperitoneal rectal cancer treated with neoadjuvant (chemo)radiotherapy plus total mesorectal excision between 2004 and 2015 were included in the study. LLND was performed when pretreatment imaging documented enlarged LLNs (7 mm or greater in size). Localization of nodal metastases and long‐term outcomes were analysed. Kaplan–Meier analysis was used to compare the survival of patients with ypN0 disease with that of patients with mesorectal ypN+/LLN− status and patients with positive LLNs. The Cox proportional hazards model was used to evaluate predictors of disease‐free survival (DFS) and local recurrence.

**Results:**

A total of 613 patients were included in the study; LLND was performed in 212 patients (34·6 per cent) and 57 (9·3 per cent) had LLN metastasis. Patients with LLN metastasis had improved DFS and local recurrence cumulative incidence rates compared with patients with mesorectal ypN2+/LLN− disease (DFS: *P* = 0·014; local recurrence: *P* = 0·006). Although the DFS rate of patients with LLN metastasis was worse than that of patients with ypN0 disease (*P* < 0·001), the cumulative incidence of local recurrence was similar (*P* = 0·491). In multivariable analysis, residual LLN metastasis was not an independent predictor of worse DFS or local recurrence.

**Conclusion:**

LLN metastasis is not an independent predictor of local recurrence or survival. Survival of patients presenting with LLN metastasis after (chemo)radiotherapy was intermediate between that of patients with ypN0 status and those with mesorectal ypN2 positivity.

## Graphical abstract

The prognostic significance of residual lateral lymph node metastasis following neoadjuvant (chemo)radiotherapy and lateral lymph node dissection in patients with advanced low rectal cancer remains unknown. This study shows that residual lateral lymph node metastasis after (chemo)radiotherapy can be considered an intermediate‐risk disease, similar to residual mesorectal ypN1 disease.

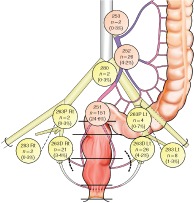

Lateral nod dissection and rectal cancer

## Abstract (es)

**Antecedentes:**

No existen en la literatura grandes estudios dirigidos a investigar la importancia pronóstica de las metástasis en los ganglios linfáticos laterales (*lateral lymph nodes*, LLN) después de la disección de los mismos (*LLN dissection*, LLND) en pacientes con cáncer de recto. El objetivo de este estudio fue evaluar el impacto pronóstico de las metástasis en los LLN sobre la supervivencia de los pacientes con cáncer de recto.

**Métodos:**

Se analizaron 613 pacientes consecutivos con cáncer de recto localmente avanzado extraperitoneal y no metastásico tratados con (quimio)radioterapia neoadyuvante seguida de resección total del mesorrecto (*total mesorectal excision*, TME) entre 2004 y 2015. Se realizó una LLND cuando el estudio mediante pruebas de imagen previo el tratamiento mostró LLN aumentados de tamaño ≥ 7 mm. Se analizó la localización de las metástasis ganglionares y los resultados a largo plazo. El análisis de supervivencia se realizó mediante el método de Kaplan‐Meier para comparar las supervivencias de los pacientes ypN0 frente a los pacientes ypN con positividad mesorrectal/LLN negativos y frente a los pacientes LLN positivos. Se utilizó el modelo de riesgo proporcional de Cox para evaluar los factores predictivos de supervivencia libre de enfermedad y de recidiva local.

**Resultados:**

Se realizó una LLND en 212 (34,6%) pacientes, y 57 (9,3%) pacientes presentaban metástasis en los LLN. Los pacientes con metástasis en los LLN presentaron mejores curvas de incidencia acumulada de recidiva local y de supervivencia libre de enfermedad en comparación con los pacientes con ganglios mesorrectales ypN2 positivos/LLN negativos (respectivamente, *P* = 0,0135 y *P* = 0,0060). Aunque la curva de la supervivencia libre de enfermedad de los pacientes con metástasis en los LLN fue peor que la de los pacientes ypN0 (*P* < 0,0001), la incidencia acumulada de recidiva local fue similar (*P* = 0,4905). En el análisis multivariable, la metástasis residual en los LLN no fue un factor predictivo independiente de peor supervivencia libre de enfermedad ni de recidiva local.

**Conclusión:**

Las metástasis en los LLN no es un factor predictivo independiente de recidiva local o supervivencia. Los pacientes que presentaron metástasis en los LLN después de (quimio)radioterapia mostraron características de supervivencia intermedias entre ypN0 y pacientes con ganglios mesorrectales ypN2 positivos.

## Introduction

Neoadjuvant (chemo)radiotherapy ((C)RT) followed by total mesorectal excision (TME) is currently the standard treatment for locally advanced rectal cancer^1^. However, previous studies from Eastern countries^2^, ^3^, ^4^ have reported that lateral lymph node (LLN) metastases outside the field of TME occurred in 10–20 per cent of patients with stage II–III extraperitoneal rectal cancer.

A recent prospective multicentre RCT (JCOG0212)^5^ compared TME with TME plus ‘prophylactic’ LLN dissection (LLND) in patients without enlargement of LLNs undergoing upfront surgery, and found that TME with LLND resulted in significantly lower local recurrence rates compared with TME alone (7·4 *versus* 12·6 per cent respectively). In contrast, in Western countries, neoadjuvant (C)RT protocols are mostly employed, resulting in local recurrence rates of less than 10 per cent^6^, with minimal indication for LLND^7^. Recently, however, Eastern^8^, ^9^, ^10^ and Western^11^, ^12^, ^13^, ^14^, ^15^ studies have suggested that (C)RT and TME without LLND may not be sufficient in patients with enlarged LLNs, with a lateral pelvic recurrence rate of 19·5 per cent in patients with LLNs 7 mm or more in size^15^.

However, large‐scale studies investigating the prognostic impact of pathologically residual LLN metastasis after (C)RT are sparse. Most previous studies focusing on LLN metastasis did not employ neoadjuvant (C)RT protocols, and poor disease‐free survival (DFS) and high local recurrence rates were also found after LLND in patients with pathological LLN metastasis^2^, ^3^, ^4^. Accordingly, residual LLN metastases are considered a feature of high‐risk tumours^16^, ^17^.

The aim of this study was to investigate the prognostic impact of LLN metastasis in patients with rectal cancer treated with neoadjuvant (C)RT and TME.

## Methods

All consecutive patients with locally advanced extraperitoneal rectal cancer without distant metastasis, treated by (C)RT and TME at the Cancer Institute Hospital (between July 2004 and December 2015) or Toranomon Hospital (between April 2010 and December 2015), were reviewed retrospectively for inclusion in the study. Exclusion criteria included the presence of inguinal metastases, owing to their rarity and the different route of lymphatic spread^18^.

In these hospitals, neoadjuvant (C)RT followed by TME is the standard treatment for locally advanced (cT3–4 or cN+) extraperitoneal rectal cancer. LLND is performed only when pretreatment imaging shows enlarged LLNs (7 mm or more in long‐axis diameter), regardless of the size of the LLNs after neoadjuvant treatment (selective LLND)^19^.

Clinical and pathological variables reviewed included: patient demographics (age, sex), tumour features (distance from anal verge, clinical stage, histological type), neoadjuvant and adjuvant protocols, surgical procedures and pathological data (ypT and ypN category according to the seventh edition of the AJCC Cancer Staging Manual^20^, nodal harvest, R status, lymphovascular invasion and grading).

Ethical approval for the study was obtained at each institution (Cancer Institute Hospital: approval number 2018‐1045; Toranomon Hospital: approval number 1711).

### Neoadjuvant (chemo)radiotherapy

Pretreatment clinical stage was assessed by CT and/or MRI in all patients. All patients received fluorouracil‐based long‐course (C)RT (total dose 45–50·4 Gy) or short‐course radiotherapy (5 × 5 Gy) with or without induction chemotherapy (mFOLFOX6 (modified folinic acid–fluorouracil–oxaliplatin regimen) or CAPOX (capecitabine–oxaliplatin) with or without bevacizumab) following evaluation by a multidisciplinary team.

The radiation field generally included all gross disease as well as the internal iliac and obturator nodes, and the perirectal and presacral spaces with the upper border at the sacral promontory. The external iliac and common iliac areas were generally not included in the radiation field. Radiotherapy was administered using the three‐field technique with the patient in the prone position.

### Lateral lymph node metastasis

Lymph nodes (LNs) were classified and numbered according to the second English edition of the Japanese Classification of Colorectal Carcinoma^21^. Mesorectal LNs included classification numbers 251 (perirectal nodes), 252 (inferior mesenteric trunk nodes) and 253 (inferior mesenteric nodes). LLNs included 263D (distal internal iliac nodes), 263P (proximal internal iliac nodes), 283 (obturator nodes), 293 (external iliac nodes), 273 (common iliac nodes) and 280 (aortic bifurcation nodes). The location of LNs was recorded by the surgeon, according to guidelines set by the Japanese Society for Cancer of the Colon and Rectum^21^.

### Lateral lymph node dissection

The LLND procedure (open or laparoscopic) has been standardized in both hospitals, and was introduced into Toranomon Hospital in April 2010^22^. LLND is performed primarily by laparoscopy (*Video* *S1* and *Appendix* *S1*, supporting information). Briefly, both internal iliac and obturator areas are dissected *en bloc*, and common iliac and aortic bifurcation areas are dissected only when metastasis is suspected, as determined by pretreatment imaging.

### Follow‐up

The follow‐up protocol included: measurement of serum carcinoembryonic antigen every 3 months for the first 3 years and every 6 months thereafter, and CT (chest to pelvis) every 6 months for 5 years. Patients who experienced no events were censored at the final follow‐up.

### Outcome measures

Localization of nodal metastases and long‐term outcomes were analysed. DFS was defined as the date of first surgery to the date on which disease recurrence (local or distant) was first detected, or death from any cause, as described previously^23^, ^24^. Local recurrence was defined as any anastomotic, pelvic or perineal tumour recurrence, diagnosed radiologically, histologically or clinically. Lateral pelvic recurrence was defined as recurrence in the lateral pelvic region.

### Statistical analysis

Continuous variables are expressed as median (i.q.r.) values, and compared with the Mann–Whitney *U* test. Survival analysis was performed using the Kaplan–Meier method, with the log rank test to compare survival of patients with ypN0 status *versus* mesorectal ypN1+/LLN− patients *versus* mesorectal ypN2+/LLN− patients *versus* LLN+ patients. Predictors of DFS and local recurrence were evaluated using the Cox proportional hazards model and included the following co‐variables: age, sex, distance from anal verge, grading, ypT category, LLN metastasis, mesorectal nodal metastasis, lymphovascular invasion and adjuvant chemotherapy. Results were reported using hazard ratios (HRs) with 95 per cent confidence intervals. Analyses were performed using GraphPad Prism® 7 software (GraphPad, San Diego, California, USA) or JMP® software V10.0.2 (SAS Institute, Cary, North Carolina, USA).

## Results

**Figure 1:**
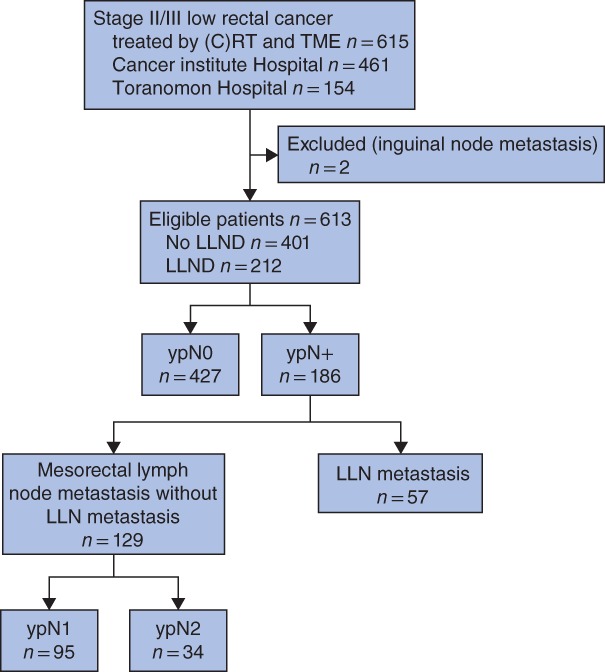
Flow diagram for the study (C)RT, (chemo)radiotherapy; TME, total mesorectal excision; LLND, lateral lymph node dissection; LLN, lateral lymph node.

A total of 615 patients were reviewed; two patients were excluded as they had inguinal LN metastasis, leaving 613 patients eligible for data analysis (*Fig*. 1). *Table* 1 shows clinical and pathological features of the selected cohort. LLND was performed in 212 patients (34·6 per cent).

**Table 1:** Patient and tumour characteristics

	No. of patients* (*n* = 613)
**Age (years)** †	61 (52–68)
**Sex ratio (M** : **F)**	415 : 198
**Distance from anal verge (mm)** †	40 (30–50)
**Clinical stage**	
I	3 (0·5)
II	213 (34·7)
III	397 (64·8)
**Histological type**	
Well/moderately differentiated	568 (92·7)
Other	45 (7·3)
**(Chemo)radiotherapy regimen**	
Long course	520 (84·8)
Short course	93 (15·2)
**Induction systemic chemotherapy**	94 (15·3)
**Interval from completion of long‐course CRT to surgery (days)** †	48 (44–53)
**Interval from completion of short‐course RT to surgery (days)** †	11 (9–17)
**Operative procedure**	
Sphincter preserving	442 (72·1)
Sphincter non‐preserving	171 (27·9)
**LLND**	
Overall	212 (34·6)
Unilateral	176 (83·0)
Bilateral	36 (17·0)
**Laparoscopic procedure**	575 (93·8)
**ypT status** ‡	
ypT0	95 (15·5)
ypT1	36 (5·9)
ypT2	179 (29·2)
ypT3	279 (45·5)
ypT4	24 (3·9)
**No. of harvested lymph nodes** †	18 (14–24)
**R status**	
R0	606 (98·9)
R1	7 (1·1)
**Lymphovascular invasion**	333 (54·3)
**Adjuvant chemotherapy**	292 (47·6)

* With percentages in parentheses unless indicated otherwise;

† values are median (i.q.r.).

‡ Residual tumor depth, according to the AJCC Cancer Staging Manual^20^. CRT, chemoradiotherapy; RT, radiotherapy; LLND, lateral lymph node dissection.

**Figure 2:**
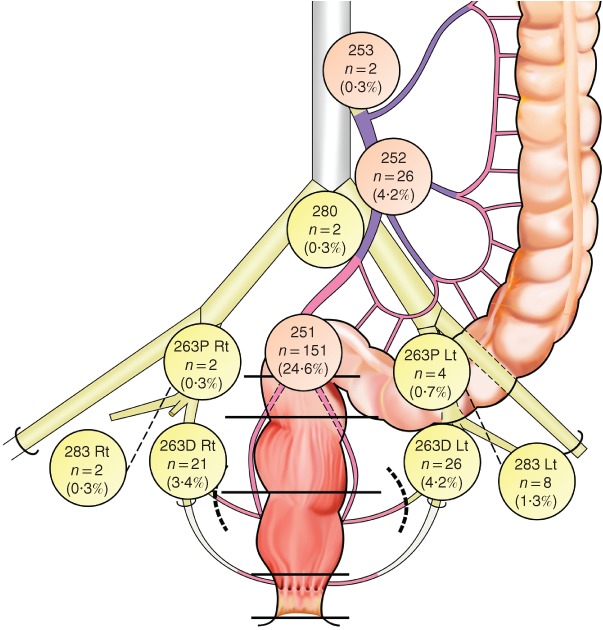
Distribution of residual lymph node metastasis after (chemo)radiotherapy in patients with advanced low rectal cancer Five patients had residual lateral lymph node metastasis in at least two different lateral areas, and three had bilateral residual lateral lymph node metastasis. Rt, right; Lt, left.

Of the 613 patients, 129 (21·0 per cent) had exclusively mesorectal LN metastasis (ypN1, 95; ypN2, 34) and 57 patients (9·3 per cent) had LLN metastasis (*Fig*. 1). Of the 57 patients with LLN metastasis, 26 (46 per cent) had exclusively LLN metastasis and 31 (54 per cent) had both mesorectal and LLN metastasis. *Fig*. 2 shows the locations of the LN metastases. The most common site of LLN metastasis was the distal internal iliac nodes (263D). The rate of LLN metastasis was higher than that of inferior mesenteric trunk node metastasis (site 252: 4·2 per cent).

### Nodal metastases

The median number of LLN metastases per patient was 1 (range 1–4). Of the 57 patients with LLN metastasis, 41 patients (72 per cent) had one metastatic node, ten (18 per cent) had two metastatic nodes, and the remaining six had three or more nodal metastases. In contrast, among the 160 patients with mesorectal LN metastasis, the median number of mesorectal LN metastases per patient was 2 (range 1–13) (*P* < 0·001). Sixty‐two patients (39 per cent) had one mesorectal LN metastasis, 36 (22 per cent) had two mesorectal LN metastases and 62 (39 per cent) had three or more mesorectal LN metastases. Among the 57 patients with residual LLN metastasis, 39 (68 per cent) had three or fewer total (mesorectal and lateral) LN metastases, whereas 18 (32 per cent) had four or more.

### Local recurrence and survival

**Figure 3:**
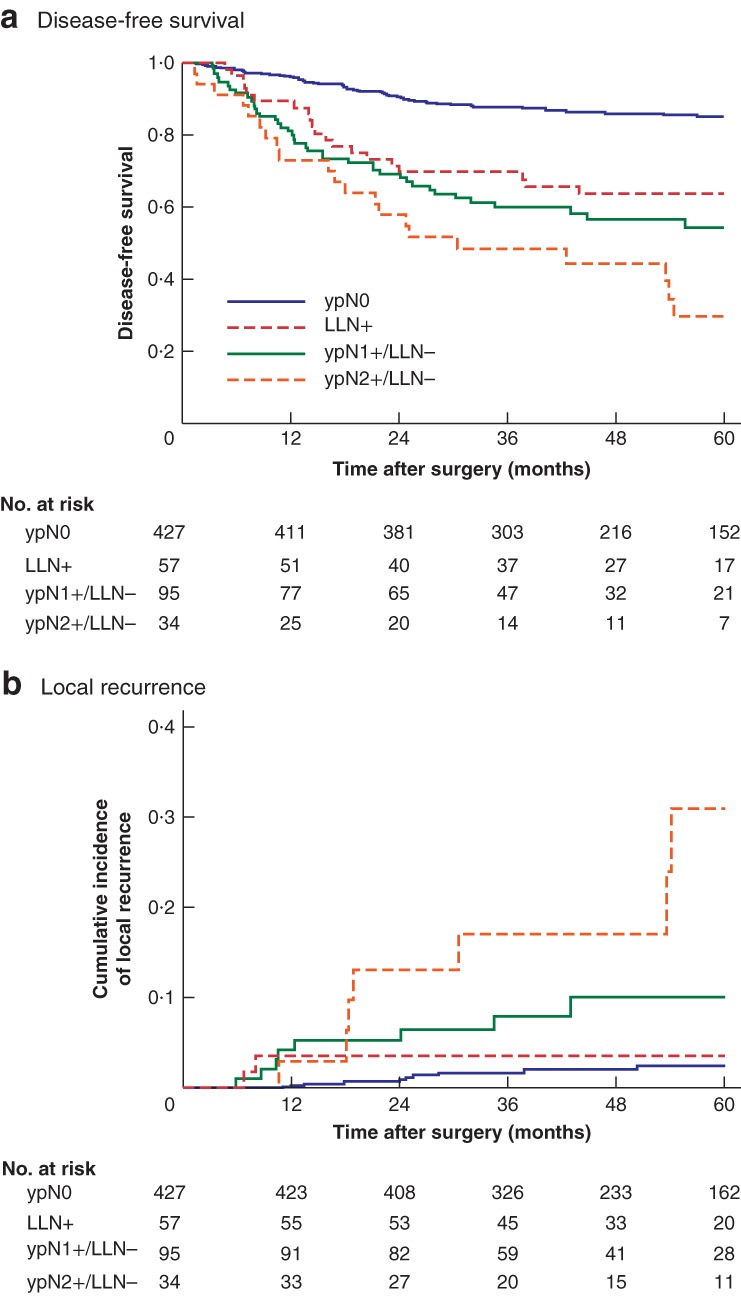
Kaplan–Meier analysis of disease‐free survival and local recurrence in patients with advanced low rectal cancer, showing the long‐term prognostic significance of ypN status after neoadjuvant (chemo)radiotherapy **a** Disease‐free survival and **b** cumulative incidence of local recurrence according to ypN metastasis status. LLN, lateral lymph node. **a** *P* < 0·001 (all groups), *P* < 0·001 (ypN0 *versus* LLN+), *P* = 0·014 (LLN+ *versus* ypN2+/LLN−); **b** *P* < 0·001 (all groups), *P* = 0·006 (LLN+ *versus* ypN2+/LLN−) (all log rank test).

Median follow‐up was 51·4 (i.q.r. 36·8–66·4) months. DFS and the cumulative incidence of local recurrence according to the pathological nodal status (ypN) are summarized in *Fig*. 3. Twenty‐six patients had local recurrence, of whom 20 (77 per cent) had lateral pelvic recurrence. Of these 20 patients, five (25 per cent) underwent unilateral LLND and 15 (75 per cent) did not have LLND (ypN0, 4; ypN1, 5; ypN2, 6) in relation to: the absence of enlarged LLNs (less than 7 mm in long‐axis diameter) on pretreatment imaging (13 patients); refusal to undergo LLND despite the presence of enlarged LLNs (9 × 8 mm) (1 patient); and undetected enlarged LLNs (9 × 9 mm) (1 patient), noted on a second imaging revision. Of the five patients who underwent LLND, four (80 per cent) without LLN metastasis had ipsilateral lateral pelvic recurrence and one patient (20 per cent) with LLN metastasis had contralateral lateral pelvic recurrence.

DFS was significantly worse in patients with residual LLN metastasis than in those with ypN0 status (3‐year DFS rate: 70 *versus* 88 per cent respectively; *P* < 0·001), but improved in comparison with that in mesorectal ypN2+/LLN− patients (70 *versus* 48 per cent respectively; *P* = 0·014). The cumulative incidence of local recurrence in patients with LLN metastasis was significantly improved compared with that in mesorectal ypN2+/LLN− patients (3‐year local recurrence: 3·6 *versus* 17 per cent respectively; *P* = 0·006). No significant difference in the cumulative incidence of local recurrence was found for patients with LLN metastasis *versus* patients with ypN0 disease (3·6 *versus* 1·7 per cent respectively; *P* = 0·491) or those with mesorectal ypN1+/LLN− status (3·6 *versus* 8·0 per cent; *P* = 0·224).

In multivariable analysis, local recurrence was independently associated with distance from the anal verge of 40 mm or less (HR 2·76, 95 per cent c.i. 1·18 to 7·22; *P* = 0·019), ypT3–4 status (HR 3·33, 1·05 to 13·07; *P* = 0·040) and mesorectal LN metastasis (1–3 LNs: HR 4·35, 1·06 to 12·99; 4 or more LNs: HR 7·67, 2·35 to 25·17; *P* = 0·001), whereas worse DFS was independently associated with distance from the anal verge of 40 mm or less (HR 1·50, 1·07 to 2·14; *P* = 0·020), ypT3–4 status (HR 2·78, 1·79 to 4·41; *P* < 0·001), mesorectal LN metastasis (1–3 LNs: HR 3·36, 2·17 to 5·20; 4 or more LNs: HR 4·10, 2·37 to 6·98; *P* < 0·001) and no adjuvant chemotherapy (HR 1·72, 1·16 to 2·55; *P* = 0·007) (*Table* 2). The presence of residual LLN metastasis was not independently associated with DFS or local recurrence.

**Table 2:** Univariable and multivariable Cox proportional hazards analysis of clinicopathological factors for disease‐free survival and local recurrence

	Disease‐free survival				Local recurrence			
	Univariable analysis		Multivariable analysis		Univariable analysis		Multivariable analysis	
	HR	*P*	HR	*P*	HR	*P*	HR	*P*
**Age (years)**		0·061		0·547		0·581		0·942
< 70	1·00 (reference)		1·00 (reference)		1·00 (reference)		1·00 (reference)	
≥ 70	1·45 (0·98, 2·10)		1·13 (0·75, 1·68)		1·30 (0·48, 3·06)		1·04 (0·35, 2·68)	
**Sex**		0·521		0·376		0·748		0·969
M	1·00 (reference)		1·00 (reference)		1·00 (reference)		1·00 (reference)	
F	0·89 (0·61, 1·27)		0·85 (0·58, 1·22)		1·14 (0·49, 2·06)		1·02 (0·42, 2·31)	
**Distance from anal verge (mm)**		0·037		0·020		0·033		0·019
> 40	1·00 (reference)		1·00 (reference)		1·00 (reference)		1·00 (reference)	
≤ 40	1·423 (1·02, 2·01)		1·50 (1·07, 2·14)		2·44 (1·07, 6·26)		2·76 (1·18, 7·22)	
**Grading**		0·005		0·220		0·040		0·378
Well/moderate (G1–2)	1·00 (reference)		1·00 (reference)		1·00 (reference)		1·00 (reference)	
Other (G3–4)	2·15 (1·28, 3·42)		1·39 (0·81, 2·27)		3·18 (1·06, 7·81)		1·65 (0·51, 4·42)	
**ypT status**		< 0·001		< 0·001		< 0·001		0·040
≤ ypT2	1·00 (reference)		1·00 (reference)		1·00 (reference)		1·00 (reference)	
ypT3–4	4·08 (2·80, 6·11)		2·78 (1·79, 4·41)		5·99 (2·29, 20·47)		3·33 (1·05, 13·07)	
**Lateral LN metastasis**		0·042		0·992		0·766		0·132
No	1·00 (reference)		1·00 (reference)		1·00 (reference)		1·00 (reference)	
Yes	1·69 (1·02, 2·64)		1·00 (0·59, 1·61)		0·81 (0·13, 2·72)		0·37 (0·06, 1·30)	
**Mesorectal LN metastasis**		< 0·001		< 0·001		< 0·001		0·001
No	1·00 (reference)		1·00 (reference)		1·00 (reference)		1·00 (reference)	
1–3	3·49 (2·41, 5·03)		3·36 (2·17, 5·20)		4·85 (1·95, 12·20)		4·35 (1·06, 12·99)	
≥ 4	5·13 (3·18, 8·00)		4·10 (2·37, 6·98)		8·96 (3·20, 24·06)		7·67 (2·35, 25·17)	
**Lymphovascular invasion**		< 0·001		0·337		0·004		0·826
No	1·00 (reference)		1·00 (reference)		1·00 (reference)		1·00 (reference)	
Yes	3·02 (2·08, 4·50)		1·25 (0·80, 1·99)		3·61 (1·47, 10·80)		1·14 (0·38, 4·01)	
**Adjuvant chemotherapy**		0·376		0·007		0·352		0·468
No	1·00 (reference)		1·00 (reference)		1·00 (reference)		1·00 (reference)	
Yes	1·16 (0·84, 1·61)		0·58 (0·39, 0·86)		1·44 (0·67, 3·22)		0·70 (0·28, 1·84)	

Values in parentheses are 95 per cent confidence intervals. HR, hazard ratio; LN, lymph node.

## Discussion

Previous studies^8^, ^9^, ^10^ have documented high rates of lateral pelvic recurrence in patients with enlarged LLNs not treated by LLND after (C)RT. One study^8^ of 116 patients with ypN+ disease reported lateral pelvic recurrence rates of 35·7 and 87·5 per cent respectively when enlarged LLNs had a short‐axis diameter of 5–9·9 mm and 10 mm or more. Similar data have been reported from the West, with a recent study^11^ finding a lateral local recurrence rate of 33·3 per cent at 4 years when LLNs had a short‐axis diameter of 10 mm or above. A recent multi‐institutional international retrospective study^15^ from 12 institutions reported that patients with LLNs and a short‐axis diameter of at least 7 mm had a significantly higher risk of lateral local recurrence than patients with LLNs of less than 7 mm. In addition, a recent survey^25^ showed that most radiation oncologists in the USA treat involved LLNs with curative intent and recommend treatment intensification, in the form of LLND, radiotherapy boost, or both. Collectively, these studies indicate the need to consider a standardized treatment approach.

Although patients with LLNs had a better outcome than those with mesorectal ypN2+ disease, a previous multi‐institutional study^2^ from Japan showed that the survival rate of patients with LLN metastasis was similar to that of patients with pN2 status when neoadjuvant (C)RT was not performed. Importantly, LLND was highly standardized in the present series with *en bloc* lymphadenectomy; a previous study^15^ in this field showed little benefit from limiting resection to the affected LLNs, as more than half of the patients later developed local recurrence in the same compartment.

LLND was probably unnecessary in 73·1 per cent of the patients. Furthermore, six patients with ypN2 status without LLN metastasis had lateral pelvic recurrence even though they showed no enlarged LLNs on imaging. Therefore, further studies are necessary to determine the optimal indication for LLND after (C)RT.

LLND is a technically challenging procedure in obese patients, and quality control, formal training with structured courses or proctoring^13^ might be necessary for international uptake of LLND.

Limitations of this study include the relatively shorter interval from (C)RT to surgery and the lower rate of induction chemotherapy compared with modern Western standards, which may have affected the rate of LLN metastasis. Second, patients were treated in high‐volume centres and most of the Japanese institutions do not use neoadjuvant (C)RT routinely.

A prospective randomized trial comparing TME alone *versus* TME with LLND after (C)RT for patients with enlarged LLNs could be difficult to design, considering the low percentage of patients with residual LLN metastasis.

## Supporting information


**Video S1.** Lateral lymph node dissection

**Appendix S1.** Procedure for lateral lymph node dissection
