# Supplementary material for: Prognostic impact of residual lateral lymph node metastasis after neoadjuvant (chemo)radiotherapy in patients with advanced low rectal cancer
Source: BJS Open. 2019 Jul 25;3(6):822–9. doi: 10.1002/bjs5.50194 (PMC6887909; doi:10.1002/bjs5.50194)

**BJS5_50194**

**Prognostic impact of residual lateral lymph node metastasis after neoadjuvant (chemo)radiotherapy in patients with advanced low rectal cancer**

**T. Akiyoshi, S. Toda, T. Tominaga, K. Oba, K. Tomizawa, Y. Hanaoka, T. Nagasaki, T. Konishi, S. Matoba, Y. Fukunaga, M. Ueno and H. Kuroyanagi**

**Video S1** Lateral lymph node dissection

**Appendix S1** Procedure for lateral lymph node dissection

Patient was placed in the lithotomy position under general anesthesia. Five ports were placed as follows: a 12-mm port at the umbilicus for the scope, and 5-mm or 12-mm ports at the bilateral upper and lower abdominal quadrants. The patient was then placed in a steep Trendelenburg position to displace the small bowel out of the pelvis. The ureter was isolated and displaced away from the left side. The external iliac artery and vein were exposed. Dissection was performed along the surface of the major psoas and internal obturator muscles. The lateral side of the umbilical artery was dissected, and the surface of the bladder was exposed. The obturator nerve was identified and preserved, but the obturator vessels were divided. The root of the internal iliac artery was clipped and divided because metastatic lateral pelvic lymph nodes had adhered to the internal iliac artery. The inferior vesical artery and vein were divided at the entrance to the bladder. The sacral plexus was exposed, and the internal iliac vein was also clipped and divided. Distal sides of the internal iliac artery/vein (internal pudendal or inferior gluteal artery/vein) were divided.


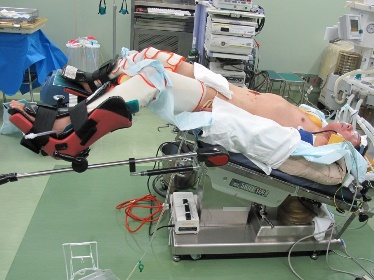
　　　　
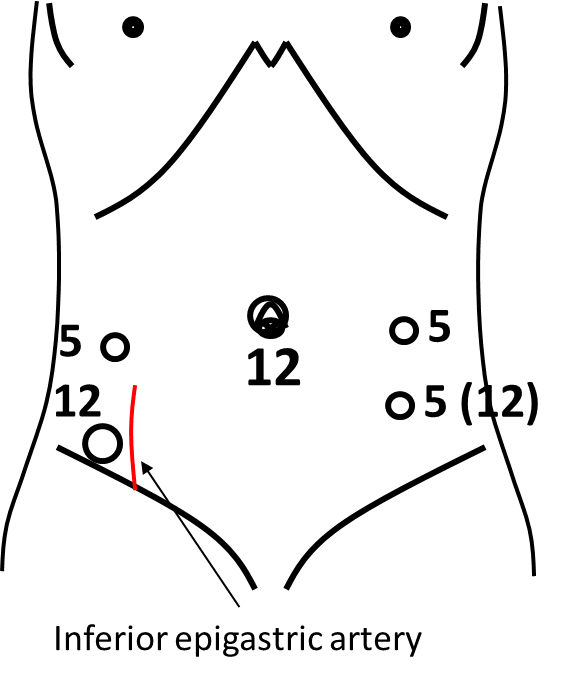

Supplement: Supplementary file 2 — Appendix S1. Procedure for lateral lymph node dissection [file BJS5-3-822-s002.docx]
